# A novel negative-stranded RNA virus mediates sex ratio in its parasitoid host

**DOI:** 10.1371/journal.ppat.1006201

**Published:** 2017-03-09

**Authors:** Fei Wang, Qi Fang, Beibei Wang, Zhichao Yan, Jian Hong, Yiming Bao, Jens H. Kuhn, John H. Werren, Qisheng Song, Gongyin Ye

**Affiliations:** 1 State Key Laboratory of Rice Biology & Ministry of Agriculture Key Lab of Molecular Biology of Crop Pathogens and Insects, Institute of Insect Sciences, Zhejiang University, Hangzhou, China; 2 Analysis Center of Agrobiology and Environmental Sciences & Institute of Agrobiology and Environmental Sciences, Zhejiang University, Hangzhou, China; 3 National Center for Biotechnology Information, National Library of Medicine, National Institutes of Health, Bethesda, Maryland, United States of America; 4 Integrated Research Facility at Fort Detrick (IRF-Frederick), Division of Clinical Research (DCR), National Institute of Allergy and Infectious Diseases (NIAID), National Institutes of Health (NIH), Fort Detrick, Frederick, Maryland, United States of America; 5 Department of Biology, University of Rochester, Rochester, New York, United States of America; 6 Division of Plant Sciences, College of Agriculture, Food and Natural Resources, University of Missouri, Columbia, Missouri, United States of America; University College London, UNITED KINGDOM

## Abstract

Parasitoid wasps are important natural enemies of arthropod hosts in natural and agricultural ecosystems and are often associated with viruses or virion-like particles. Here, we report a novel negative-stranded RNA virus from a parasitoid wasp (*Pteromalus puparum*). The complete viral genome is 12,230 nucleotides in length, containing five non-overlapping, linearly arranged open reading frames. Phylogenetically, the virus clusters with and is a novel member of the mononegaviral family *Nyamiviridae*, here designated as Pteromalus puparum negative-strand RNA virus 1 (PpNSRV-1). PpNSRV-1 is present in various tissues and life stages of the parasitoid wasp, and is transmitted vertically through infected females and males. Virus infections in field populations of *P*. *puparum* wasps ranged from 16.7 to 37.5%, without linearly correlating with temperature. PpNSRV-1 increased adult longevity and impaired several fitness parameters of the wasp, but had no influence on successful parasitism. Strikingly, PpNSRV-1 mediated the offspring sex ratio by decreasing female offspring numbers. RNA interference knockdown of virus open reading frame I eliminated these PpNSRV-1-induced effects. Thus, we infer that PpNSRV-1 has complex effects on its insect host including sex ratio distortion towards males, as well as possible mutualistic benefits through increasing wasp longevity.

## Author summary

Although a few viruses with RNA genomes have been discovered in parasitoid wasps, their numbers are limited and their effects on the wasps have rarely been determined. Unambiguous negative-sense, single-stranded RNA [(-)ssRNA] viruses in parasitoids wasps have not been described. In this report, we identify a novel (-)ssRNA virus from a parasitoid wasp, verify the presence of virion-like particles in various wasp tissues, and characterize the viral genomic structure, transcription strategy, phylogenetic relationship, transmission strategy, and biological impacts on its host. Most importantly, we reveal that this novel virus mediates secondary sex ratio of its host by decreasing female offspring numbers, and can be transmitted both by male and female to offspring. Decrease in female offspring could be due to increased female mortality or to alterations in the primary sex ratio. The reduced female offspring number per female could therefore be a fecundity cost or a tradeoff for increased longevity of the wasp. However, it remains to be determined whether increased longevity under laboratory conditions translates into increased lifetime fecundity for the wasp in nature. Increased longevity could be beneficial to the virus by promoting its transmission to more hosts. The secondary sex-ratio distortion induced by the virus may also increase its transmission in host populations, because males transmit the virus to their offspring and can mate with multiple females, thus further spreading the virus.

## Introduction

Parasitoid wasps (Hymenoptera: Aprocrita) are important natural enemies of arthropods (Ecdysozoa: Arthropoda) and are widely used as bio-control agents against insect (Arthropoda: Hexapoda: Insecta) pests in agro-ecosystems. They are frequently associated with viruses or virion-like particles (VLPs). To date, viruses of seven families have been identified in parasitoid wasps: double-stranded DNA (dsDNA) viruses (*Ascoviridae*, *Polydnaviridae*, *Poxviridae*), positive-sense, single-stranded RNA [(+)ssRNA] viruses (*Coronaviridae*, *Iflaviridae*), and segmented double-stranded RNA [dsRNA] viruses (*Reoviridae*) [1]. Among these viruses, polydnaviruses are most often detected in parasitoids wasps [2] and they are the best known example of an insect/virus symbiosis. Each polydnavirus from a wasp of a given species persists as an integrated provirus in the germ line and somatic cells of the wasp’s body. Polydnaviruses are therefore endogenous virus elements (EVEs) that have become genetically fixed in different wasp lineages. When polydnavirions are injected into the body of the wasp’s lepidopterous host, viral DNAs can be discharged into the cell nuclei and integrated into the genome of infected host cells [3]. The expression products of polydnaviral genes are exclusively beneficial to parasitoid wasps for successful survival and emergence from their hosts by suppressing the host immune system and modifying host growth, development, and metabolism [4]. Similarly, Diachasmimorpha longicaudata entomopoxvirus (DlEPV) infects host hemocytes and induces cytopathic effects that disable host encapsulation to promote the wasp offspring survival [5]. In contrast, Diadromus pulchellus ascovirus 4a (DpAV4), vectored by *Itoplectis tunetana* wasps, prevents the development of parasitoid eggs and larvae by quickly triggering cell lysis [6].

In addition, VLPs devoid of DNA or RNA have been observed in ovarian tissue or the venom apparatus of parasitoid wasps, and their functions are well studied [7–11]. For example, the unclassified filamentous Leptopilina boulardi filamentous virus (LbFV) alters superparasitism behavior and parasitism rates of the infected parasitoid wasp (*Leptopilina boulardi*) by increasing its tendency to lay supernumerary eggs in parasitized hosts [12–15]. The transmission of LbFV is wasp density-dependent, which may influence the coexistence of distinct leptopilines. For instance, wasps of the species *L*. *boulardi* can quickly outcompete wasps of the species *L*. *heterotoma* in the absence of LbFV, whereas wasps of the species *L*. *heterotoma* can maintain or even eliminate those of the species *L*. *heterotoma* in the presence of LbFV [16].

Although a few RNA viruses have been discovered in parasitoid wasps, their effects on the wasps have rarely been determined [1]. With the recent adoption of new next generation sequencing (NGS) technologies, several new (+)ssRNA viruses have been discovered in parasitoid wasps. For example, three novel (+)ssRNA viruses could be identified in cDNA libraries of *Nasonia vitripennis* wasps. Two of these (+)ssRNA viruses, (Nasonia vitripennis viruses 1 and 2 [NvitV-1/2]) probably belong to the family *Iflaviridae* in the order *Picornavirales*; whereas the third virus (NvitV-3) is most similar to the unclassified *Picornavirales* member Nora virus, which infects fruit flies [17]. However, the effect of these viruses on their hosts remains unknown. Recently, a new iflavirus named Dinocampus coccinellae paralysis virus (DcPV) has been found in *Dinocampus coccinellae* wasps, which parasitize the lady beetle (*Coleomegilla maculate*). The replication of DcPV in lady beetle cerebral ganglia most likely induces changes in lady beetle behavior, such as tremors, gait disturbance, and limitations in movement [18].

Nonsegmented negative-sense single-stranded (-)ssRNA viruses, i.e. viruses of the order *Mononegavirales*, are of considerable importance and include notorious human, animal, and plant viral pathogens, such as filoviruses, paramyxoviruses, and rhabdoviruses [19]. Most mononegaviruses have been found in vertebrates and plants whereas only a few mononegaviruses have been detected in invertebrates (S1 Table): soybean cyst nematode virus 1 (SbCNV-1, *Nyamiviridae*: *Socyvirus*) was found in soybean cyst nematodes, and is phylogenetically closely related to tick-borne nyaviruses (*Nyamiviridae*: *Nyavirus*) [20]. In insects, rhabdoviral sigmaviruses were discovered in drosophilids [21]. Flies infected with sigmaviruses become paralyzed or die after exposure to high concentrations of CO_2_, whereas uninfected flies can recover. Similar symptoms can also be found in parasitized mosquitos [22] and aphids [23].

Only one (-)ssRNA virus, Diachasmimorpha longicaudata rhabdovirus (DlRhV), has been found in parasitoid wasps (*Diachasmimorpha longicaudata*) [24]. However, no evidence is available to confirm that the DlRhV genome is indeed related to rhabdoviruses, and the role of this virus in the successful development of its host wasp has not been clearly illuminated [1].

Members of the species *Pteromalus puparum* are predominantly pupal parasitoid wasps that prey on butterflies of several species including the small white (*Pieris rapae*), with the highest parasitism rate over 90% reported in fields of cruciferous vegetables in China [25]. *P*. *puparum* wasps are gregarious and synovigenic parasitoids. The vitellogenesis of *P*. *puparum* wasps is initiated after the pupal stage and completed at the adult stage 48 h after eclosion [26]. Adult nutrition significantly affects ovarian development [27]. Unlike parasitoid wasps carrying polydnaviruses, this parasitoid wasp has evolved a different means of using venom to suppress the host immune system and modify host development [28, 29]. The composition and functions of the venom from this parasitoid have been well studied by our laboratory [30–36]. To better understand the relationship between this parasitoid wasp and its hosts, we recently sequenced the wasp’s transcriptome and discovered a novel virus. We determined the complete viral genome and confirmed that this virus, named Pteromalus puparum negative-strand RNA virus 1(PpNSRV-1), belongs to the mononegaviral family *Nyamiviridae*, but is unrelated to viruses of the existing nyamiviral genera *Nyavirus* and *Socyvirus*. We further investigated the tissue and developmental expression profile, field prevalence, transmission strategy, and biological characteristics of PpNSRV-1. The most striking discoveries are that PpNSRV-1 is vertically transmitted by both infected female and male wasps, and that the virus modifies the secondary sex ratio of the parasitoid host by reducing female offspring number. This is the first report of an unambiguous mononegavirus in parasitoid wasps.

## Results

### PpNSRV-1 is a novel virus encoding at least five proteins

**Fig 1:**
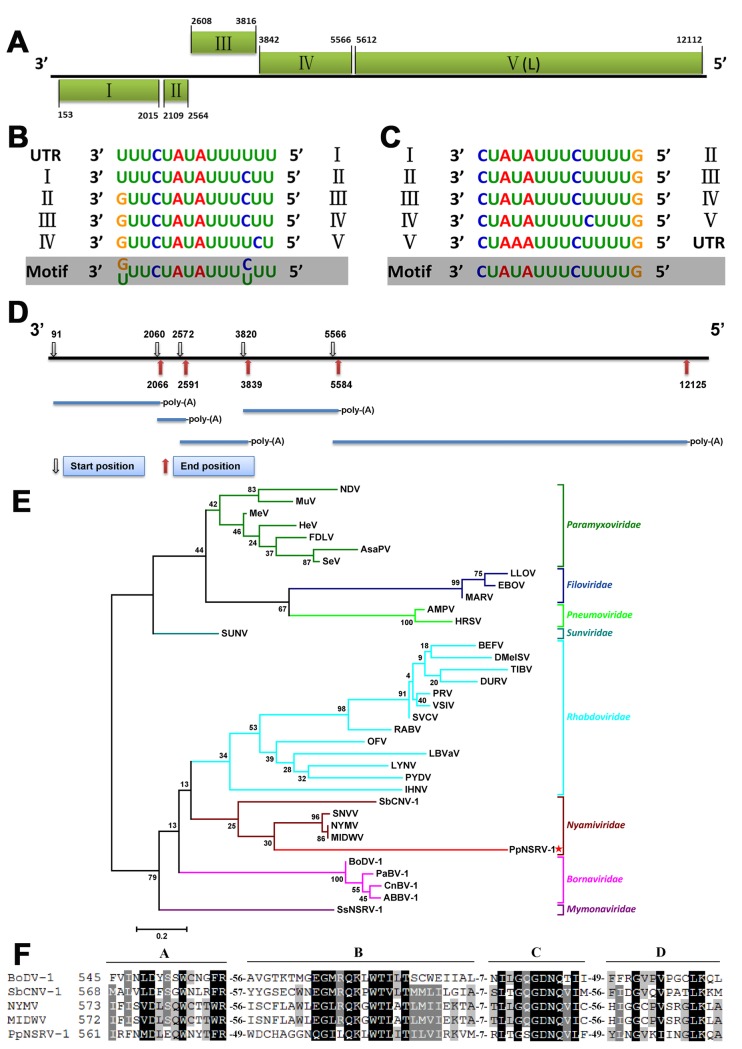
Genomic organization of PpNSRV-1. (A) Genome length and organization of PpNSRV-1. Open reading frame (ORF) I and ORF II are in the same frame, ORF IV and ORFV are in another frame, and ORF III is in third frame. Boxes indicate the position and length of each ORF. ORF V encodes the RNA-dependent RNA polymerase (RdRp, L). (B) Putative transcription initiation sequences. (C) Putative transcription termination sequences. The consensus motif is shown below the sequences for each viral ORF in a 3′ to 5′ orientation. (D) The possible transcript map of PpNSRV-1 based on 5′ and 3′ RACE. (E) Phylogenetic analysis of PpNSRV-1: phylogram of the core RdRp motif of PpNSRV-1 and selected viruses from all mononegaviral families. The position of PpNSRV-1 is indicated with a red star. Bootstrap values in 1,000 replications are shown. (F) Multiple sequence alignment of the core RdRp motif of PpNSRV-1 and selected mononegaviruses. Identical residues are shaded in black and similar residues are in grey. Conserved motifs are marked A to D. Numbers at the beginning of the sequences represent the amino acid positions from the N-terminus of the L protein. Abbreviations of virus names and viral protein accession numbers are the same as those listed in S1 Table.

Analysis of the transcriptome of the parasitoid wasp *Pteromalus puparum* uncovered a large contig (12,141 bp) containing regions similar to viral RNA-dependent RNA polymerases (RdRps). Consequently, we sequenced the associated viral genome, including the 5′ and 3′ genome termini. The complete genome of PpNSRV-1 is 12,230 nucleotides in length (Genbank #KX431032). G+C pairs comprise 46.09% of the nucleotides. The PpNSRV-1 genome contains five large open reading frames (ORFs I–V) located at nucleotide (nt) positions 153 to 2015, 2109 to 2564, 2608 to 3816, 3842 to 5566, and 5612 to 12112, respectively. The leader and trailer regions of the PpNSRV-1 genome are 152 and 118 nt in length, respectively (Fig 1A). Their terminal nucleotides are not complementary.

By comparing the 5′ and 3′ untranslated regions and intergenic regions of the PpNSRV-1 genome, we pinpointed the putative transcription initiation and termination signals for each of the five ORFs. A conserved transcription initiation motif of 3′-(G/U)UUCUAUAUUU(C/U)UU-5′ was identified upstream of each putative ORF at 4–44 nt before the start codon (Fig 1B). Similarly, the transcription termination motif of 3′-CUAUAUUUCUUUUG-5′ was detected downstream of every ORF at 1–104 nt downstream of the stop codon (Fig 1C). No intergenic, non-transcribed regions were noted between transcription initiation and termination motifs. To further elucidate the transcriptional strategy of PpNSRV-1, primers were designed for rapid amplification of cDNA ends (RACE) of each ORF. RACE results indicated that all five ORFs can be transcribed independently as shown in the transcript map (Fig 1D). The virus lacks a poly-(A) tail at 3′ terminus.

### PpNSRV-1 is a mononegavirus

We confirmed transcription of the five ORFs of the PpNSRV-1 genome by northern blot analysis (S1 Fig). General properties of all ORFs are listed in Table 1. The predicted translation product of PpNSRV-1 ORF V was found to be similar in amino-acid sequence to that of the Midway virus RNA-dependent RNA polymerases. Predicted products of the other four ORFs are not similar to any deposited protein sequence. Computational sequence analyses were performed to identify potential functional motifs in the predicted PpNSRV-1 proteins. Only ORF IV protein was predicted to be a putative type I transmembrane protein and to possess a cleavable signal peptide. Moreover, ORF IV protein contains seven potential *O*-linked and four potential *N*-linked glycosylation sites and 29 potential phosphorylation sites, indicating that ORF IV encodes the viral glycoprotein (mononegaviral G ortholog/analog). Based on the conserved genomic orientation of mononegaviruses (3′-N-P-M-G-L-5′), PpNSRV-1 ORF II, containing nine phosphorylation sites, most likely encodes the phosphoprotein (P). ORF III, containing 37 potential *O*-linked glycosylation sites, may encode a glycosylated matrix protein (M) and ORF I consequently would encode the nucleoprotein (N).

**Table 1:** Calculated and Predicted Properties of PpNSRV-1 ORFs.

Viral ORF	ORF length (nt)	Protein length (aa)	Protein mass (kDa)	pI	Signal peptide	No. of glycosylation sites		No. of phosphorylation sites			Top BLASTX match	E value
						*O*-linked	*N*-linked	Ser	Thr	Tyr		
**ORF I**	1863	620	69.13	5.47	-	11	0	15	16	6	ND	-
**ORF II**	456	151	16.86	5.45	-	15	0	6	3	0	ND	-
**ORF III**	1209	402	43.5	8.73	-	37	0	24	15	2	ND	-
**ORF IV (G)**	1725	574	63.36	6.39	+	7	4	16	10	3	ND	-
**ORF V (L)**	6501	2166	245.88	7.24	-	15	0	43	24	16	RdRp (Midway virus)	2E-83

ND, no significant homology was detected.

### PpNSRV-1 represents a novel nyamiviral genus

The most closely related known sequence to PpNSRV-1 ORF V is the RNA-dependent RNA polymerase (L) of Midway virus (*Mononegavirales*: *Nyamiviridae*: *Midway nyavirus*). Therefore we conducted a maximum likelihood phylogenetic analysis of the amino acid core sequences of the PpNSRV-1 ORF V protein to determine the relationship of PpNSRV-1 to other mononegaviruses. Representatives of each official genus of the seven official mononegaviral families [19] were included in the phylogenetic tree. Notably, PpNSRV-1 clusters as a distinct lineage in family *Nyamiviridae* (Fig 1E, brown), indicating the need for a novel virus species in a novel nyamiviral genus. Multiple alignments of the predicted core RdRp motifs of PpNSRV-1 ORF V protein with those of other mononegaviruses revealed the mononegavirus-typical four highly distinct and conserved motifs (A to D) (Fig 1F).

Pairwise Sequence Comparison (PASC) analysis of the PpNSRV-1 genome revealed that it is 13.8% identical to the genome of soybean cyst nematode virus 1 (SbCNV-1; nyamiviral genus *Socyvirus*), 12.3% identical to that of Nyamanini virus (NYMV; nyamiviral genus *Nyavirus*), 12.1% identical to that of Midway virus (MIDWV; nyamiviral genus *Nyavirus*), and 10.9% identical to that of Sierra Nevada virus (SNVV; nyamiviral genus *Nyavirus*), with those viruses being the closest known relatives of PpNSRV-1. This value (13.8% identity between the PpNSRV-1 and SbCNV-1 genome) is lower than the 17% identity measured between nyavirus and socyvirus genomes, indicating the need for a novel nyamiviral genus.

### PpNSRV-1 tissue titers are highest in adult female wasps

**Fig 2:**
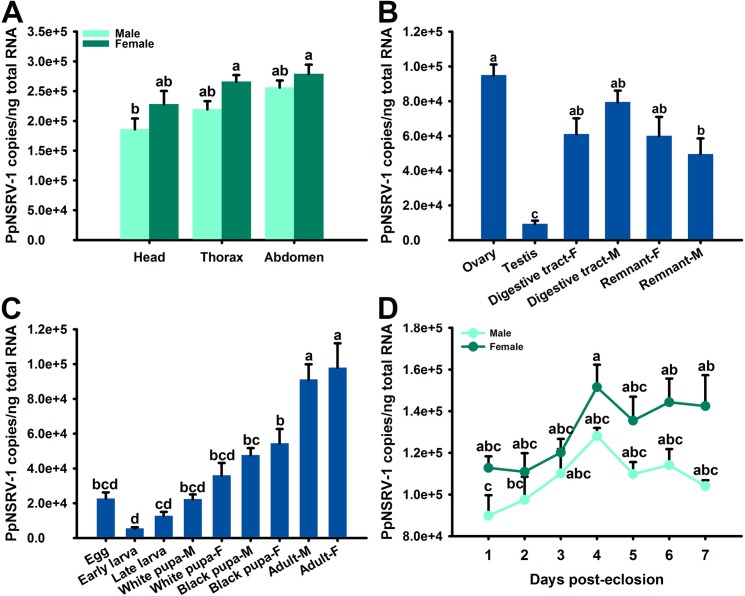
Viral load of PpNSRV-1 in different tissues and developmental stages of *P*. *puparum* wasps. (A) Viral load of PpNSRV-1 in head, thorax, and abdomen, (B) in ovaries, testes, female tract (tract-F), male tract (tract-M), female remnant (remnant-F), and male remnant (remnant-M), and (C) in eggs, larvae, pupae and adult wasps. (D) Viral load in adult wasps from 1 day to 7 day post-eclosion. Data represent means ± standard error (*n* = 3). SE bars annotated with the same letters are not significantly different (Tukey’s multiple comparison test).

The PpNSRV-1 titers in the head, thorax, and abdomen of infected *P*. *puparum* wasps as analyzed by qPCR were not significantly different (*F* = 1.088, *df* = 2, *p* = 0.3680, Tukey’s multiple comparison test, Fig 2A). However, the female body segments contained more viral RNA compared to male body segments (*F* = 18.123, *df* = 1, *p* = 0.0011, Fig 2A). Tissue specific distribution analysis revealed significant differences among ovaries, testes, digestive tracts and female or male body remnant (*F* [5, 12] = 13.588, *p* = 0.0001). The highest viral titer was found in ovaries, followed by digestive tract and body remnant. Testes had the lowest viral titer (Fig 2B).

PpNSRV-1 was detected in the eggs, larvae, pupae and adults of *P*. *puparum* wasps by developmental expression analysis. The viral titer increased significantly from the larval to adult stages (*F* (8, 18) = 21.935, *p* <0.0001) and peaked in adults (Fig 2C). PpNSRV-1 genome copy number was the highest at days 4 post-eclosion (Fig 2D) (Male: [*F* [6, 14] = 2.251, *p* = 0.0990] and Female: [*F* [6, 14] = 2.497, *p* = 0.0743]). At higher environmental temperatures (35°C versus 25°C), viral titers in both female and male wasps decreased marginally but not significantly (Female: [*F* = 0.263, *df* = 1, *p* = 0.6151] and Male: [*F* = 0.284, *df* = 1, *p* = 0.6313]) (S2A and S2B Fig).

### PpNSRV-1 antigen is widely distributed in wasp tissues

**Fig 3:**
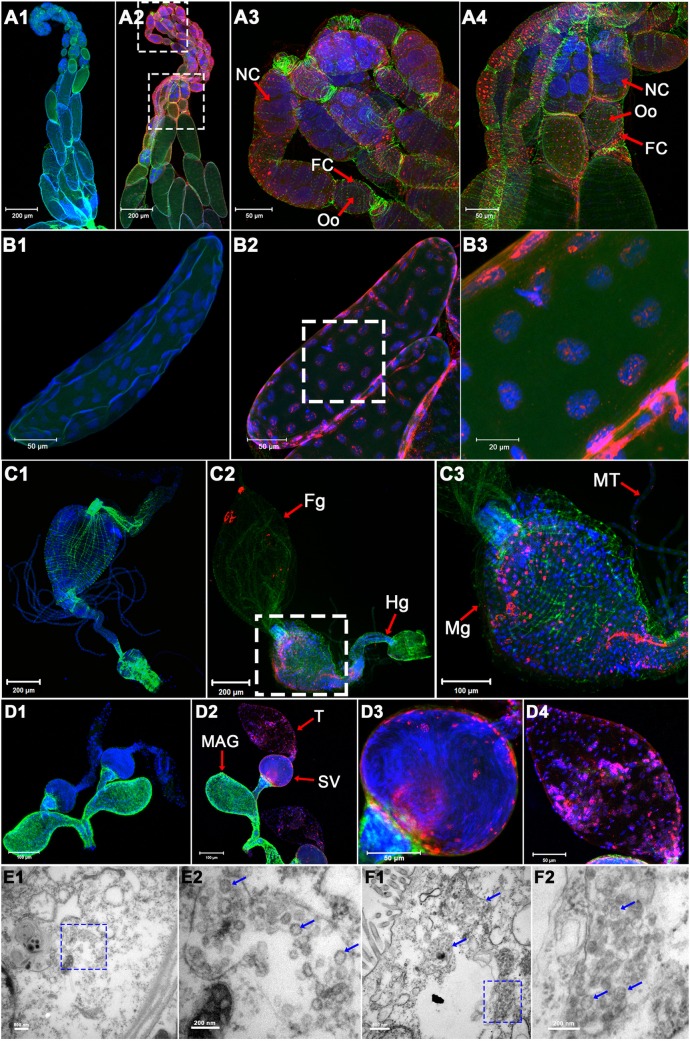
Immunohistochemical visualization and electron micrographs of PpNSRV-1 antigen and particles. (A) Immunolocalization of PpNSRV-1 antigen in ovary. A3 and A4 are the enlarged insets of the boxes in A2. (B) Immunolocalization of PpNSRV-1 antigen in eggs. B3 is the enlarged inset of the box in B2. (C1–C3) Immunolocalization of PpNSRV-1 antigen in digestive tract. B3 is the enlarged inset the box in B2. (D) Immunolocalization of PpNSRV-1 antigen in sperm. D3 and D4 are the enlarged insets of the box in D2. (A1, B1, C1, and D1) and (A2, B2, C2, and D2) were dissected from PpNSRV-1(-) and PpNSRV-1(+) wasps, respectively. F-actin was stained with phalloidin (green). Cell nucleus was stained with DAPI (blue). PpNSRV-1 virion clusters were detected by use of a rabbit anti-PpNSRV-1 polyclonal antibody and goat anti-rabbit IgG labeled with red-fluorescent secondary antibody. (E) Virions observed in ovaries as indicated by blue arrows on electron micrographs. E2 is the enlarged inset of the box in E1. (F) Virions observed in digestive tract as indicated by blue arrows on electron micrographs. F2 is the enlarged inset of the box in F1. NC: nurse cells; Oo: oocyte; FC: follicle cells; Fg: foregut; Mg: midgut; Hg: hindgut; MT: malpighian tubule; T: testis; SV: seminal vesicle; MAG: male accessory gland. Blue arrows indicated viral particles.

Immunohistochemistry revealed PpNSRV-1 antigen in ovaries (Fig 3A2–3A4), eggs (Fig 3B2 and 3B3), midgut (Fig 3C2 and 3C3), testes and seminal vesicles (Fig 3D2–3D4) of the PpNSRV-1-infected (PpNSRV-1 (+)) wasps. In the ovarioles, the viral signals were much higher in oocytes, follicle cells, and the intracellular space compared to nurse cells (Fig 3A3 and 3A4). PpNSRV-1 antigen could not be detected in tissues sampled from uninfected PpNSRV-1 (PpNSRV-1(-)) wasps (Fig 3A1, 3B1, 3C1, and 3D1).

### PpNSRV-1 particles resemble those of nyaviruses

To study PpNSRV-1 particle morphology, we prepared sections of the digestive tract and ovaries from PpNSRV-1(+) wasps and of purified particles and examined by transmission electron microscopy (TEM). Spherically shaped VLPs (62.5–100 nm in diameter) were present in follicular cells of the ovaries (Fig 3E1 and 3E2 and S3 Fig). Similarly, VLPs (70–100 nm in diameter) stacked in intracellular vesicles were observed in cells of the digestive tract (Fig 3F1 and 3F2). VLPs were not found in PpNSRV-1(-) wasps. The spherical PpNSRV-1 particle shape is similar to particles produced by nyaviruses, i.e. MIDWV and NYMV.

### PpNSRV-1 is transmitted vertically by its host wasp

**Fig 4:**
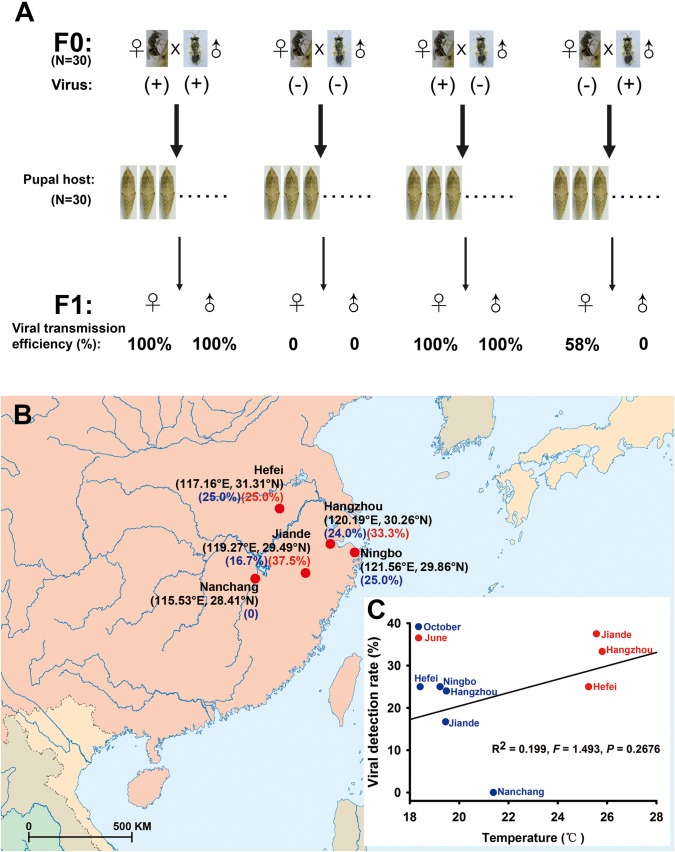
Vertical transmission of PpNSRV-1 in *P*. *puparum* wasps and viral detection in field populations of *P*. *puparum* wasps. (A) Vertical transmission of PpNSRV-1 to offspring. In the parent wasps, (-) means virus-free and (+) means virus-infected. The N value is the sample size for each treatment. (B) Viral detection in wild populations of *P*. *puparum* wasps from parasitized butterfly pupae collected from different cities in China. In the wasps collected in October 2012 and June 2016, the infection rate (%) is shown in blue and red, respectively. (C) Relationship between viral detection rate and mean temperature from the five different locations sampled in October 2012 and June 2016.

PpNSRV-1 is vertically transmitted from both PpNSRV-1(+) females and males to their offspring, although the patterns differ between the sexes (Fig 4A and S4B–S4E Fig). Notably, transmission was 100% from infected females to both their male and female progeny, but transmission from infected males when the mate is uninfected occurred only to female offspring (58% to female progeny but 0% to male progeny, Fig 4A and S4E Fig).

### PpNSRV-1 is present in parasitoid wasps in geographic disparate areas

Field *P*. *puparum* wasps were collected from five different locations in China to investigate the prevalence of PpNSRV-1 in different geographic locations. The virus was detected in four wasp populations sampled from Ningbo, Hangzhou, Jiande (all Zhejiang Province), and Hefei (Anhui Province) with the PpNSRV-1-positive rate ranging from 16.7% to 37.5%. PpNSRV-1 prevalence decreased with dropping latitude, with no virus detection in samples collected from the lowest latitude location in Nanchang (Jiangxi Province). However, the PpNSRV-1-positive rate was neither significantly correlated with temperature (Fig 4C, R^2^ = 0.199, *F* = 1.493, *p* = 0.2676, Tukey’s multiple comparison test), nor with latitude in October (R^2^ = 0.740, *F* = 8.536, *p* = 0.0614, Tukey’s multiple comparison test). In contrast, PpNSRV-1 could not be detected in unparasitized butterfly pupae collected from all five locations (S3 Table).

### PpNSRV-1 influences the offspring sex ratio of its parasitoid host wasp

**Fig 5:**
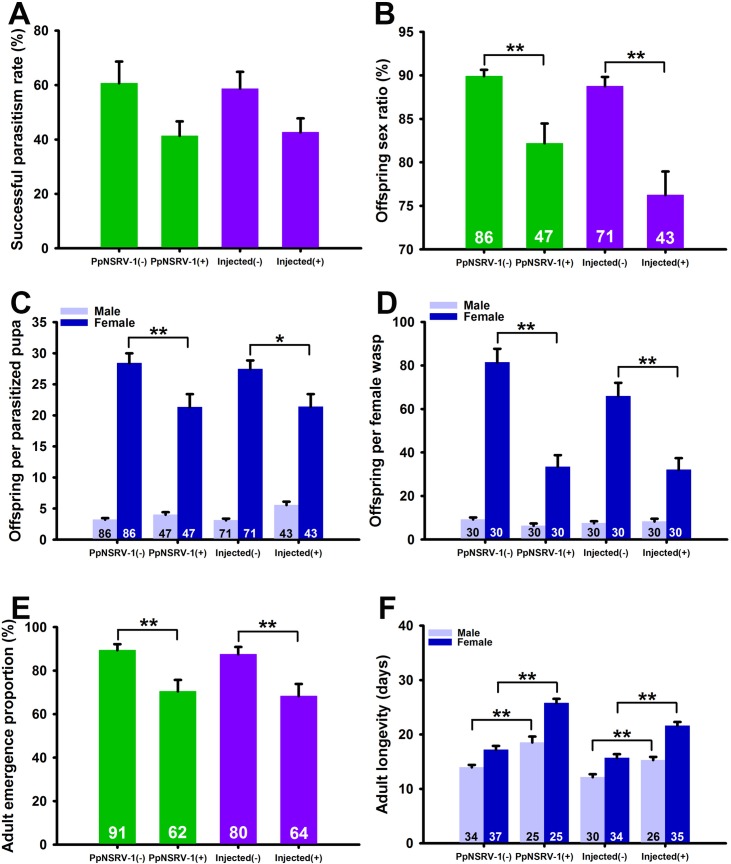
Effect of PpNSRV-1 on key biological parameters of *P*. *puparum* wasps. The effect of PpNSRV-1 on successful parasitism rate (A), offspring sex ratio (B), offspring per parasitized pupa (C), offspring per female wasp (D), adult emergence proportion (E), and adult longevity (F) among wild colonies and injected pupae. (-) means virus-free and (+) means virus-infected. The number in the column is the sample size for each treatment. Data represent means ± standard error. Statistical significance (*t*-tests) is indicated by asterisks: *, *p* < 0.05; **, *p* < 0.01.

Comparison of the PpNSRV-1(+) and PpNSRV-1(-) wasp colonies revealed no significant difference in the successful parasitism rate (*t* = 2.013, *df* = 5, *p* = 0.0789, *t*-test, Fig 5A) and the total oocyte number (*t* = 1.591, *df* = 28, *p* = 0.1229, S5 Fig). However, offspring sex ratio (*t* = 3.959, *df* = 131, *p* = 0.0001, Fig 5B), female offspring number emerging per parasitized pupa (*t* = 2.682, *df* = 131, *p* = 0.0083, Fig 5C), female offspring number laid by per female wasp (*t* = 5.833, *df* = 58, *p* <0.0001, Fig 5D), and adult emergence proportion (*t* = 3.434, *df* = 151, *p* = 0.0008, Fig 5E) were significantly lower in the PpNSRV-1(+) colony compared to the PpNSRV-1(-) colony. Interestingly, the longevity of both female and male wasps in the PpNSRV-1(+) colony was significantly longer than compared to that of the PpNSRV-1(-) colony (female: *t* = 7.936, *df* = 60, *p* <0.0001; male: *t* = 4.239, *df* = 57, *p* = 0.0001, Fig 5F).

Similarly, under higher environmental temperature (35°C), the longevity of both female and male wasps in the PpNSRV-1(+) colony was still significantly longer compared to that of the PpNSRV-1(-) colony (female: *t* = 2.192, *df* = 73, *p* = 0.0316; male: *t* = 2.781, *df* = 76, *p* = 0.0068, (S2C Fig). Additionally, the female offspring number laid by each female wasp per day for the PpNSRV-1(+) colony was significantly less compared to that of the PpNSRV-1(-) colony in most cases (S6D Fig). However, a significantly less female offspring number per parasitized pupa per day, which was parasitized by a female, was found in the PpNSRV-1(+) colony compared to that observed in PpNSRV-1(-) control colony on the first batch of parasitism. Female offspring from the two colonies from subsequent batches of parasitism was not significantly different (S6A Fig).

To eliminate the effect of different genetic backgrounds and to confirm effects of PpNSRV-1 on parasitoid wasp biology, we injected the virus into yellow pupae from the PpNSRV-1(-) wasps (PpNSRV-1(+) group), and injected mock virus into another group as the control (PpNSRV-1(-) group). Similar results were obtained for both groups (Fig 5 and S6C and S6G Fig). Comparison of the injected (+) and injected (-) wasp colonies revealed no significant difference in the successful parasitism rate (*t* = 1.9931, *df* = 8, *p* = 0.0814, *t*-test, Fig 5A). Conversely, offspring sex ratio (*t* = 4.976, *df* = 112, *p* < 0.0001, Fig 5B), female offspring number per parasitized pupa (*t* = 2.515, *df* = 112, *p* = 0.0133, Fig 5C), female offspring number laid by per female wasp (*t* = 4.163, *df* = 58, *p* = 0.0001, Fig 5D), and adult emergence proportion (*t* = 3.060, *df* = 142, *p* = 0.0026, Fig 5E) were significantly lower in the injected (+) colony compared to the injected (-) colony. The longevity of both female and male wasps in the injected (+) colony was significantly longer than that of the injected (-) colony (female: *t* = 6.0387, *df* = 67, *p* <0.0001; male: *t* = 3.867, *df* = 54, *p* = 0.0003, Fig 5F). PpNSRV-1 titer increased steadily with wasp development after virus injection (*F* (5, 12) = 38.041, *p* <0.0001) (S7 Fig). On the 3rd day after adult eclosion, the viral load in the injected females (8.911 × 10^4^ copies/ng of total RNA) and males (8.417 × 10^4^ copies/ng of RNA, S7 Fig) was similar to the viral load in the PpNSRV-1(+) wasps (1.202 × 10^5^ copies/ng in females and 1.010 × 10^5^ copies/ng in males) (Fig 2D).

**Fig 6:**
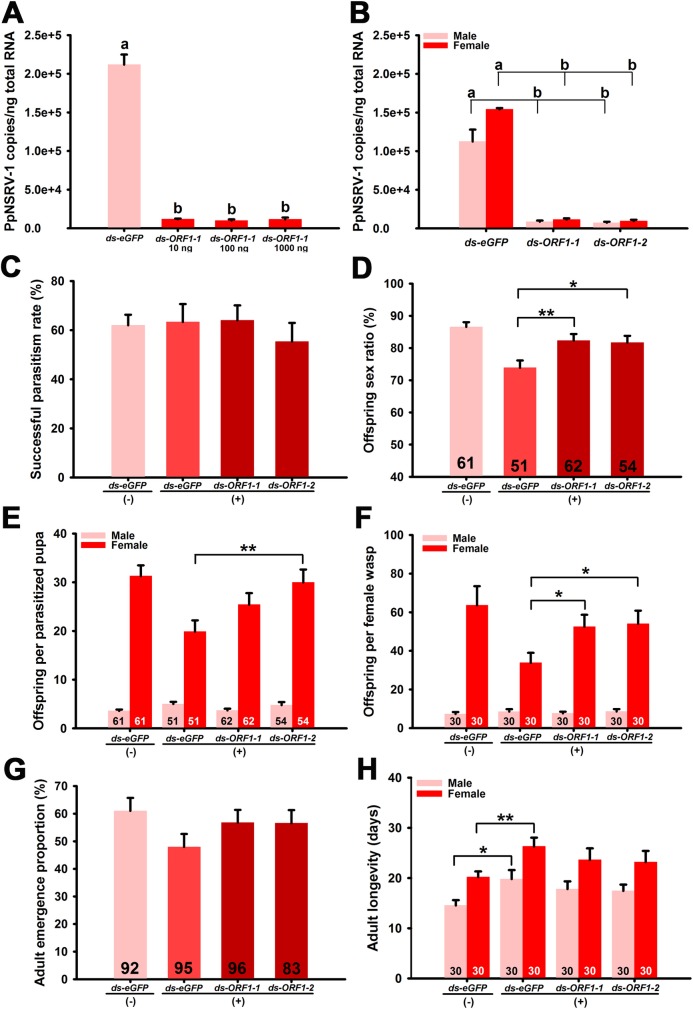
Effect of RNAi knockdown and PpNSRV-1 on key biological parameters of *P*. *puparum* wasps. (A, B) Effect of RNAi knockdown of PpNSRV-1 on virus titer as detected by qPCR in wasps 4^th^ day post-eclosion. Data represent means ± standard error (*n* = 3). SE bars annotated with the same letter are not significantly different (Tukey’s multiple comparison test). Comparison of successful parasitism rate (C), offspring sex ratio (D), offspring per parasitized pupa (E), offspring per female wasp (F), adult emergence proportion (G), and adult longevity (H) among RNAi-treated wasps. The number in column is the sample size for each treatment. Data represent means ± standard error. Statistical significance (*t*-tests) is indicated by asterisks: *, *p* < 0.05; **, *p* < 0.01.

In a previous study, dsRNA was injected into *Microplitis demolitor* wasp pupae to determine the effect on bracovirus degradation and specificity of dsRNA [37]. We followed this strategy using an RNA interference (RNAi) assay. We injected dsRNAs targeting PpNSRV-1 ORF I into the yellow *P*. *puparum* pupae from PpNSRV-1(+) wasps to investigate viral load, successful parasitism rate, adult emergence proportion, and offspring number. Virus load in the wasps on day 4 post-eclosion was quantified. For example, injection of 10 ng to 1 μg of “*ds-ORF1-1*” dsRNA yielded a similar level of knockdown (Fig 6A) (*F* (3, 8) = 221.216, *p* < 0.0001, Tukey’s multiple comparison test). PpNSRV-1 genome copies in *ds-ORF1-1*- and *ds-ORF1-2*-dsRNA injected wasps declined compared to the *ds-eGFP* dsRNA control in both sexes (Fig 6B) (female: *F* (2, 6) = 1216.426, *p* < 0.0001; male: *F* (2, 6) = 22.439, *p* = 0.0016). The successful parasitism rate had no significant difference among the dsRNA treated colonies (Fig 6C) (*F*(3,16) = 0.378, *p* = 0.7701). Offspring sex ratio (ds-ORF1-1(+): [*t* = 2.7661, *df* = 111, *p* = 0.0066] and ds-ORF1-2(+): [*t* = 2.5290, *df* = 103, *p* = 0.0130], Fig 6D), female offspring number per parasitized pupa (ds-ORF1-1(+): [*t* = 1.6558, *df* = 111, *p* = 0.1006] and ds-ORF1-2(+): [*t* = 2.8628, *df* = 103, *p* = 0.0051], Fig 6E), female offspring number laid by per female wasp (ds-ORF1-1(+): [*t* = 2.3281, *df* = 58, *p* = 0.0234] and ds-ORF1-2(+): [*t* = 2.3416, *df* = 58, *p* = 0.0227], Fig 6F), were significantly higher in the PpNSRV-1 dsRNA treated colonies compared to the ds-eGFP(+) colony. Adult emergence proportion in the ds-eGFP(-) colony was higher than that of ds-eGFP(+) colony (Fig 6G) (*t* = 1.9638, *df* = 185, *p* = 0.0511). The longevity of both female and male wasps in the PpNSRV-1 dsRNA treated colonies was shorter than that of the ds-eGFP(+) colony, but longer than that of the ds-eGFP(-) colony (female: *t* = 2.9338, *df* = 58, *p* = 0.0048 and male: *t* = 2.5293, *df* = 58, *p* = 0.0142, Fig 6H).

## Discussion

We detected and sequenced the genome of a novel (-)ssRNA virus, PpNSRV-1, from parasitoid wasps (*P*. *puparum*) and verified the presence of PpNSRV-1 particles in various wasp tissues. In addition, we characterized the PpNSRV-1 genome organization, its phylogenetic placement, transmission strategy, and biological impacts on its host. Our results represent the first unambiguous detection of a (-)ssRNA virus in insect parasitoids. Intriguingly, we revealed that PpNSRV-1 mediates the secondary sex ratio of its host wasp by decreasing female offspring numbers.

The arrangement of the PpNSRV-1 genome follows the typical basic five-ORF pattern (3′-N-P-M-G-L-5′) of mononegaviral genomes [38]. Indeed, ORF sequence analysis demonstrated that PpNSRV-1 ORF IV and V encoded a glycoprotein (G) and an RNA-dependent RNA polymerase (L), respectively. The functions of the other three PpNSRV-1 ORFs remain unclear at this point in time, but it is highly likely that they encode mononegaviral N, P, and M orthologs or analogs.

The spherical shape of PpNSRV-1 particles found in infected parasitoid wasp is similar to that of nyaviruses, in particular MIDWV and NYMV [39]. Phylogenetic analysis revealed that PpNSRV-1 clustered with viruses in the mononegaviral family of *Nyamiviridae*. This is a relatively novel family, which currently includes two genera, *Nyavirus* (Midway virus [MIDWV], Nyamanini virus [NYMV], Sierra Nevada virus [SNVV]) and *Socyvirus* (soybean cyst nematode virus 1 [SbCNV-1]) [19, 40]. All four known nyamiviruses have been found in ecdysozoans: MIDWV was discovered in 1966 in ixodid *Ornithodoros* (*Alectorobius*) *capensis* ticks collected in bird nests sampled on the US Midway and Kure Atolls in the Pacific Ocean and has since been found in ticks of the same species in Japan and *Ornithodoros* (*Alectorobius*) *denmarki* in Hawaii [40, 41]; NYMV was first discovered in 1957 in tick-infected cattle egrets (*Bubulcus ibis ibis*) and their fowl tampans (*Argas walkerae*) in Nyamanini Pan in the Ndumu Game Reserve, northern Natal, South Africa. NYMV has since been isolated from *Argas* ticks of various species collected in Egypt, India, Nepal, Nigeria, and Thailand [40, 42–44]; SNVV was isolated in 1975 from argasid ticks (*Ornithodoros coriaceus*) [45]; and SbCNV-1 was discovered in the US in 2010 in an inbred laboratory culture of soybean cyst nematodes (*Heterodera glycines*) [20]. PpNSRV-1 is therefore the first nyamivirus that infects insects. Our phylogenetic analysis and pairwise genome comparisons using PASC indicated that while PpNSRV-1 could be considered a nyamivirus, PpNSRV-1 is not closely related enough to the four known nyamiviruses to allow classification in the genera *Nyavirus* or *Socyvirus*. After consultation, the International Committee on Taxonomy of Viruses (ICTV) *Nyamiviridae* Study Group agreed with our assessment. We have therefore submitted an official taxonomic proposal (TaxoProp) together with the Study Group to the ICTV, proposing a novel nyamiviral genus (*Peropuvirus*) including a single species (*Pteromalus puparum peropuvirus*) for PpNSRV-1 (TaxoProp 2016.015a-dM.A.v1.Peropuvirus; https://talk.ictvonline.org). This proposal has by now been accepted by the ICTV Executive Committee and awaits ratification. The relatively low bootstrap values (Fig 1E) suggest that this classification may have to be corrected in the future once additional nyami-like and/or borna-like virus genomes become available for analysis. Indeed, the discovery of PpNSRV-1 indicated that the nyamiviral clade of mononegaviruses is in all likelihood highly diverse, given that the now five members of the family have been found in very different geographic areas (Africa, Asia, North America, and the Pacific) and distinct ecdysozoan clades (Chelicerata/Arachnida, Hexapoda/Insecta, and Nematoda).

We found PpNSRV-1 in all tissues and life stages of females and males of the PpNSRV-1 host, the *P*. *puparum* wasp. At this point, we consider the expression of PpNSRV-1 to be constitutive and not specific for any wasp tissue or developmental stage. In contrast, DlRhV was found in the venom apparatus and the subchorionic space of oviposited eggs, but not in the oviduct or pre-vitellogenic or chorionated vitellogenic ova of female DlRhV hosts (*Diachasmimorpha longicaudata* parasitoid wasps) [46]. In addition, we did not detect distinct pathologies in any of the tested tissues of PpNSRV-1-infected wasps. Therefore, PpNSRV-1 could be considered as a nonpathogenic commensal virus of *P*. *puparum* wasps.

Other (-)ssRNA viruses infecting insects, such as sigmaviruses, can be vertically transmitted through both eggs and sperm to the host offspring [47]. A vertical transmission pattern was also found in other parasitic wasp-associated RNA viruses that infect the tissues of the female parasitoid wasp host reproductive system [1].

The results from crossing experiments between PpNSRV-1(+) and PpNSRV-1(-) *P*. *puparum* wasps clearly supported the existence of vertical transmission route of PpNSRV-1 to the offspring via the infected wasp reproductive tissues. The vertical transmission pathway was also supported by detection of PpNSRV-1 antigen and visualization of PpNSRV-1 particles in wasp ovarioles, eggs, testes, and seminal vesicles. The vertical transmission efficiency of PpNSRV-1 differs from that of two sigmaviruses discovered in *Drosophila affinis* and *Drosophila obscura* fruit flies. In *D*. *affinis* flies, infected females transmitted Drosophila affinis sigmavirus (DAffSV) to 98% of their offspring, whereas males transmitted the virus to only 45% of their offspring. In *D*. *obscura* flies, infected females transmitted Drosophila obscura sigmavirus (DObsSV) to 92% of their offspring, whereas males transmitted the virus to 88% of their offspring [48]. But in *P*. *puparum* wasps, infected females transmitted PpNSRV-1 to 100% of their female and male offspring, whereas males transmitted the PpNSRV-1 to 58% of their female offspring and 0% of their male offspring. The vertical transmission mode of PpNSRV-1 may explain why four out of five field populations of *P*. *puparum* wasps have a ≈16.7–37.5% infection rate, and why our PpNSRV-1(+) colony can be stably reared in the laboratory from one generation to the next.

Especially noteworthy is that males transmit PpNSRV-1 exclusively to female offspring, as only female offspring is derived from fertilized eggs in haplodiploid insects such as *P*. *puparum* wasps. This result is intriguing, and suggests that the virus is transmitted within sperm or is tightly associated with sperm, because in haplodiploid species, sperm-fertilized eggs develop into female progeny while unfertilized eggs develop into male progeny [49]. An alternative explanation is that the virus is also integrated into the wasp’s nuclear genome and transmitted vertically through chromosomes. Further experimentation is underway to resolve these alternatives. Recently, a double-stranded RNA virus found in *L*. *boulardi* wasps (Leptopilina boulardi toti-like virus, LbTV) has a male vertical transmission pattern through the maternal lineage even if frequent paternal transmission also occurs [50]. This result suggests that the transmission pattern we observed with PpNSRV-1 may be more widespread in nature than previously appreciated.

How *P*. *puparum* wasps are infected with PpNSRV-1 remains unclear and should be investigated in future. In our studies, PpNSRV-1 prevalence in field populations was positively correlated with latitude, but was not significantly correlated with temperature. The female sex ratio of overwintering *P*. *puparum* wasp field generations was less than 50%, which we consider to be related to superparasitism or higher offspring number in a host [25, 51, 52], but could also be due to a seasonal increase in frequency of the virus.

By comparing biological parameters between PpNSRV(+) and PpNSRV(-) colonies of *P*. *puparum* wasps, we found that PpNSRV-1 can affect the offspring sex ratio of the host parasitoids by decreasing the total female offspring number. The underlying mechanism of PpNSRV-1-mediated secondary sex ratio alteration remains unclear. In arthropods, a diverse array of vertically-transmitted endosymbiotic microorganisms have been discovered that alter sex ratio or sex determination, such as bacteria of the genera *Cardinium*, *Arsenophonus*, *Spiroplasma*, *Rickettsia*, and *Wolbachia*, as well as microsporidian fungi [53, 54]. The best example are wolbachiae, which can induce different reproductive changes in arthropods, including feminization, parthenogenesis, male killing and sperm-egg incompatibility [55]. Examples that viruses change host sex ratio are a masculinizing virus in the common pill-bug (Arthropoda: Isopoda: *Armadillidium vulgare*) [56] and a male-killing virus-like RNA sequences in the oriental tea tortrix moth (*Homona magnanima*) [57]. The PpNSRV-1-mediated pattern is consistent with a female-killing phenotype. Although male-killing sex ratio distorters are common in nature [53, 58], female-killing is rare and would seem to be maladaptive for the virus, as it is transmitted vertically through females. However, unlike most other sex-ratio distorting elements, the virus is also vertically transmitted through males. It is possible that male transmission is more effective (e.g. because male mates multiply, thus increasing transmission to other families), and transmission through males can be further enhanced if uninfected females produce a secondary sex-ratio bias towards daughters, which is common in *P*. *puparum*. In this regard, spread of the virus could be enhanced in the same manner of the male-biasing supernumary chromosome *psr* in the parasitoid was *N*. *vitripennis* [59], and the masculinaizing virus in the isopod *A*. *vulgare* [56]. In both these cases, the male-distorting element is enhanced by female biased sex ratios in populations. *P*. *puparum* wasps generally produce a female-biased sex ratio[25, 51, 52, 60, 61], which is consistent with Hamilton’s local mate competition theory [62] and is a phenomenon found in many other parasitoid wasps [63, 64]. PpNSRV-1 also increases longevity of infected males and females, suggesting that the virus may provide mutualistic benefits to the wasps. Clearly, more research needs to be performed to substantiate this and other hypotheses about the effects of PpNSRV-1 on its hosts.

## Methods

### Insect rearing

The laboratory colonies of *Pteromalus*. *puparum* wasps and its butterfly hosts, the small white (*Pieris rapae*), were initially collected from cabbage fields in the experimental farmland of Zhejiang University, Hangzhou, China in 2012 and maintained as described previously [28]. The wasps that had successfully parasitized host pupae were individually tested for the presence of PpNSRV-1 using the methods described below. Offspring from a single PpNSRV-1(-) or PpNSRV-1(+) breeding pair was reared to produce the PpNSRV-1(-) or PpNSRV-1(+) colony.

### Virus genome sequencing

Primers for viral genome sequencing were designed based on the virus genome-like contig discovered during transcriptional profiling of *P*. *puparum* wasps (S2 Table). Total RNA from adult female wasps was extracted using TRIzol (Invitrogen, California, USA). RNA concentrations of each sample were measured using Nanodrop 2000 (Thermo Scientific, Wilmington, DE). Single-strand cDNA was synthesized from the RNA using the TransScript One-Step gDNA Removal and cDNA Synthesis SuperMix Kit (TransGen Biotech, Beijing, China). cDNA was used as a template for PCR. The terminal sequences of the viral genome were confirmed by 5′ or 3′ RACE according to the instructions of the SMART RACE cDNA amplification Kit (Clontech, California, USA). All amplified PCR products were cloned into pGEM-T Easy vectors (Promega (Beijing) Biotech Co., Ltd. Beijing, China) and sequenced.

### Sequencing, northern blot analysis, and phylogenetic analysis

Nucleotide sequence analysis and assembly were performed using DNAStar software version 5.02 (Madison, WI, USA). ORFs of the PpNSRV-1 genome were predicted using open-source NCBI ORF finder (https://www.ncbi.nlm.nih.gov/orffinder/). For each ORF, signal peptides and transmembrane regions were predicted using open-source Phobius web server [65]. Molecular weight and isoelectric point (pI) of the predicted ORFs were calculated via open-source ProtParam (http://web.expasy.org/protparam/). Potential phosphorylation sites were determined by open-source NetPhos 2.0 [66], and glycosylation sites were determined by NetOGlyc 4.0 [67] and NetNGlyc 1.0 (http://www.cbs.dtu.dk/services/NetNGlyc/).

To identify putative conserved transcription termination and initiation sequences of the PpNSRV-1 genome, we analyzed noncoding viral genome regions using the open-source MEME suite of motif-based sequence analysis tools [68].

Northern blot analysis was performed to confirm transcription of predicted PpNSRV-1 ORFs. Total RNA from PpNSRV-1(-) or PpNSRV-1(+) wasps were extracted as described above. Separation of RNA was performed using formaldehyde gel electrophoresis, followed by transfer to Hybond N^+^ nylon membranes (GE Healthcare) by upward capillary transfer in 20 × saline sodium citrate buffer and cross-linking by UV illumination. DNA fragments were amplified by primer pairs (S2 Table) based on the each ORF sequence and were labelled using the PCR DIG Probe Synthesis Kit (Roche, Mannheim, Germany). Hybridization was performed at 42°C and the detection was carried out using the DIG-High Prime DNA Labelling and Detection Starter Kit II according to the manufacturer’s instructions (Roche).

Multiple alignments of amino acid sequences were performed using open-source Clustal Omega (http://www.ebi.ac.uk/Tools/msa/clustalo/) and edited by open-source GeneDoc (http://www.softpedia.com/get/Science-CAD/GeneDoc.shtml). The NCBI PASC classification tool was used for pairwise comparisons of viral genomes [69]. The phylogenetic tree was constructed using the maximum likelihood method with 1000-fold bootstrap resampling using MEGA 5.05 software [70]. Accession numbers of analyzed mononegaviral genomes are listed in S1 Table.

### Virus detection and quantification

To detect PpNSRV-1 in *P*. *puparum* wasps, two pairs of primers amplifying 506 bp and 523 bp fragments, VDA-1/VDS-1 and VDA-2/VDS-2, were designed according to the genomic sequence of PpNSRV-1 (S2 Table). Total RNA from each sample was used to synthesize cDNA as described above. PCR was run as follows: 30 s at 94°C, 30 s at 55°C, and 45 s at 72°C for 35 cycles. Amplifications were visualized by 1% agarose gel electrophoresis and ethidium bromide staining.

qPCR was used to quantify the viral load of PpNSRV-1. An absolute standard curve was constructed from a plasmid clone of the corresponding PpNSRV-1 genome region using specific primers ABVA/ABVS (S2 Table). PCR products were cloned into pGEM-T Easy vectors and then sequenced. Standard curves were generated by determination of copy numbers (10^3^–10^8^ copies) of standard plasmid. qPCR was performed using the Bio-Rad CFX 96 Real-Time Detection System (Bio-Rad, Hercules, CA, USA) with SYBR *Premix Ex* Taq II (Tli RNaseH Plus) (Takara Bio, Otsu, Japan). Thermal cycling conditions were: 94°C for 30 s, 40 cycles of 95°C for 5 s, and 60°C for 30 s. Three replicates of samples of each group were performed. The equation of *y* = -0.3174*x* +12.43 (*y* = the logarithm of plasmid copy number to base 10, *x* = Ct value, R^2^ = 0.9937) was used to calculate the copy number of PpNSRV-1 genomes.

### Tissue distribution and developmental expression patterns of PpNSRV-1

Different tissues or groups of tissues from adult female and male *P*. *puparum* wasps were dissected to evaluate tissue tropism of PpNSRV-1. Female and male wasps from virus-infected colonies that were fed on 20% (v/v) honey for 5 days were placed into the glass tubes. The tubes were put on the ice, and the wasps were chilled for a few seconds. The head, thorax and abdomen of each wasp was separated using a dissecting microscope (Leica, Wetzlar, Germany) and directly removed into TRIzol for RNA extraction. The abdomina from female or male adult wasps were further dissected to gain digestive tracts, ovaries or testes. For each tissue, the PpNSRV-1 genome copy number was measured by qPCR. A pool of fifteen wasps was used for RNA extraction for each sample as one replicate, which was repeated three times. The extraction of total RNA, the synthesis of single-strand cDNA, and qPCR were conducted for each sample as described above.

To define the expression profile of PpNSRV-1 in *P*. *puparum* wasps at different developmental stages, eggs, larvae, and pupae from both female and male adults (with the age from 1 day to 7 days post-eclosion) were collected, and then placed directly into TRIzoL. To evaluate whether *P*. *puparum* wasps could clear PpNSRV-1 infection at high environmental temperature, both female and male adults (aged 1, 3, 5, or 7 days post-eclosion) were fed at 25°C or 35°C and collected to determine viral titers. For qPCR, total RNA of each developmental sample was extracted and single-strand cDNA synthesized as described above. A pool of five wasps was used for each replicate, and the experiment was repeated three times.

### Polyclonal antibody preparation

A cDNA fragment (1.9 kb) of PpNSRV-1-ORF I was amplified using primers VORF1A/VORF1S (S2 Table). Purified PCR products were cloned into vector pET-28a (+) and confirmed by DNA sequencing. The construct was used to transform *Escherichia coli* BL21 (DE3) using standard procedures. Bacterial cells were collected by centrifugation and disrupted by sonication. The insoluble recombinant His-tagged protein (72.7 kDa) was purified using Ni-chelating affinity columns (TransGen Biotech, Beijing, China) under denaturing conditions. To confirm the identity of the recombinant protein, proteins were separated by SDS-PAGE, transferred to polyvinylidene difluoride (PVDF) membranes (Sigma, St. Louis, MO) by a semi-dry electrophoretic transfer system (Bio-Rad), and detected with an anti-His monoclonal antibody conjugated to horseradish peroxidase (HRP) (HuaAn Biotechnology, Hangzhou, China) as described previously [71]. Signals were visualized with an enhanced chemiluminescence detection system (Super Signal West Pico Chemiluminescent Substrate; Pierce, Rockford, IL). The purified protein was submitted to HuaAn Biotechnology Company as an antigen for immunization in male New Zealand rabbits. The obtained polyclonal antibody to PpNSRV-1 ORF I protein was purified from antiserum by the company. The company reported an antibody titer of 1:1000.

### PpNSRV-1 antigen detection by immunohistochemistry

For immunohistochemistry, ovaries, eggs, digestive tracts and male reproductive organs from PpNSRV-1(-) or PpNSRV-1(+) wasps were dissected, washed, and handled as described previously [72]. The primary antibody was rabbit anti-PpNSRV-1-ORF I (diluted 1:100 in phosphate-buffered saline (PBS) containing 5% goat serum). The secondary antibody was DyLight 549-conjugated goat anti-rabbit (diluted 1:200 in PBS, Abbkine, Redlands, CA, USA). The actin cytoskeleton was stained by FITC-phalloidin (green; diluted 1:500, Good Biotech., Wuhan, China). The nucleus was stained with 1 μg/ml 4′-6-diamidino-2-phenylindole (DAPI [blue], Good Biotech., Wuhan, China). Samples were analyzed and images recorded using a Zeiss LSM 780 confocal microscope (Carl Zeiss SAS, Jena, Germany). A stack of consecutive confocal optical sections (Z-stacks) were recorded at 8 bit. Figures were merged and scale bars were added using Zeiss LSM ZEN 2010 software (Carl Zeiss SAS, Jena, Germany). Adobe Photoshop CC (Adobe Systems Inc., San Jose, CA) was used for image grouping. All samples were analyzed with the same microscope and software settings.

### Visualization of PpNSRV-1 particles by transmission electron microscopy

Ovaries and digestive tracts of *P*. *puparum* wasps were dissected from 5-day-old adult females using a dissecting microscope (Leica, Wetzlar, Germany). Samples of ovaries and digestive tracts were pre-fixed overnight at 4°C with 4% glutaraldehyde in phosphate buffered saline (PBS; 0.01 M, pH 7.4). Samples were washed in PBS three times every 15 min and post-fixed with 2% osmium tetroxide (OsO_4_) in PBS for 1–2 h, and washed again. Samples were dehydrated by a graded series of ethanol (30%, 50%, 70%, 80%, 90%, 95%, and 100%) for about 15 to 20 min at each step and transferred to absolute acetone for 20 min. Cells were infiltrated with a 1:1 mixture of absolute acetone and the final Spurr resin mixture for 1 h at room temperature, transferred to 1:3 mixture of absolute acetone and the final resin mixture for 3 h, and to a final Spurr resin mixture overnight. Finally, each specimen was placed in an Eppendorf tube containing Spurr resin, incubated at 70°C for more than 9 h, and then sectioned using an ultramicrotome (LEICA EM UC7, Wetzlar, Germany). Ultrathin sections were double-stained by uranyl acetate for 5 min and alkaline lead citrate for 10 min and then were observed using a transmission electron microscope (Hitachi Model H-7650 TEM, Tokyo, Japan) at an accelerating voltage of 80 kV.

To confirm virus particle morphology, we purified virus particles from PpNSRV-1(+) wasps. Two thousand adult wasps were ground, and the virus particles were purified as described previously [73]. The ground samples were dissolved in 0.1 M phosphate buffer (pH 7.0). After removing impurities by centrifugation at 12,000×*g* for 20 min at 4°C, a mixture of equal volumes of chloroform and butanol was added to the supernatant and mixed for 20 min at room temperature until the stratification disappeared. The mixture was centrifuged at 12,000×*g* for 20 min at 4°C. Virus particles resided in the upper (butanol) phase). Virus particles were precipitated by ultracentrifugation (Beckman Coulter Life Sciences, Indianapolis, IN, USA) at 33,000×*g* for 4 h at 4°C. The resulting precipitate was resuspended in 0.01 M of PBS (pH 7) and then centrifuged to remove impurities (12,000×*g* for 20 min at 4°C). The resulting virus sample was frozen at -70°C for further investigation. Purified particles were stained by phosphotungstic acid and observed using a transmission electron microscope.

### Venereal and vertical transmission of PpNSRV-1

The newly-emerged wasps from PpNSRV-1(-) and PpNSRV-1(+) colonies of *P*. *puparum* wasps were individually kept in sterilized glass tubes and fed with 20% honey solution for 24 h. For each crossing experiment, 10 pairs of female and male wasps were allowed to mate for 24 h. Both female and male wasps were then individually moved to new sterilized glass tube containers (18 × 82 mm) and reared by providing one newly-pupated small white butterflies for the female wasp to parasitize. Before emergence, female and male wasp pupae were individually dissected from parasitized butterfly pupae to avoid offspring mating. Both dissected female and male wasp pupae were individually kept in sterilized glass tubes until emergence. Once emerged, all the female or male wasps from the same parasitized host pupa were collected for virus detection separately using methods described above. Four crossed experiments were designed: female(+) × male(+), female(-) × male(-), female(+) × male(-), and female(-) × male(+). Viral transmission efficiency was calculated based on the wasps emerged from 10 parasitized host pupae as described previously [74].

### Detection of PpNSRV-1 in field populations of *P*. *puparum* wasps

To investigate the prevalence of PpNSRV-1 in field populations, we collected some parasitized pupae from five different cities of eastern China including Ningbo (121.56°E, 29.86°N, 108 pupae), Hangzhou (120.19°E, 30.26°N, 75 pupae), Jiande (119.27°E, 29.49°N, 90 pupae), Hefei (117.16°E, 31.31°N, 96 pupae), and Nanchang (115.53°E, 28.41°N, 84 pupae) in October, 2012, respectively. To determine the overall annual PpNSRV-1, we collected some parasitized pupae from Hangzhou (90 pupae), Jiande (96 pupae), and Hefei (80 pupae) in June, 2016. In the vertical transmission assay, we found that the female parents must be virus-free if the male offspring did not harbor the virus. So we detected the pool individuals to estimate the virus prevalence in the female parents. Once emerged, 5 female and 3 male wasps dissected from the same pupa were collected for virus detection using methods described above. Detection of virus in female or male wasps from the parasitized pupa was considered indicative of whole-colony infection. Prevalence was calculated as described previously [74]. Butterfly pupae not parasitized by *P*. *puparum* wasps were also collected from the five locations as mentioned above and individually tested for the presence of PpNSRV-1 using the same method described above to ensure they were uninfected.

### Effect of PpNSRV-1 on key biological parameters of *P*. *puparum* wasps

Major biological parameters including the successful parasitism rate, adult emergence proportion, offspring number and sex ratio, and longevity of *P*. *puparum* wasps were compared between the PpNSRV-1(-) and PpNSRV-1(+) colonies and calculated as described previously [60]. Total oocytes number in ovaries was calculated to compare the fecundity between PpNSRV-1(-) and PpNSRV-1(+) wasps. Using a dissecting microscope, 15 female wasps from the PpNSRV-1(-) and PpNSRV-1(+) colony were dissected to count oocytes in each ovary. 30 female wasps from the PpNSRV-1(-) and PpNSRV-1(+) colony were paired with males, allowed to mate for 48 h, and then placed individually in glass tube containers with one newly pupated butterfly. During the following 5 days, each pupa encountered by the female wasp was individually transferred into a new glass tube daily, and the newly-pupated pupa and cotton ball containing honey solution (20%) was replaced. After the wasp offspring emerged, both female and male offspring from each pupa were collected, counted, individually moved into different glass tubes, and fed with 20% honey solution in cotton balls. The number of dead female or male adult wasps was counted daily, and the life span (from emergence to death) of each adult was calculated. Meanwhile, wasps that failed to emerge from parasitized pupa were also counted after peeling off the pupae. To check whether PpNSRV-1 could kill wasps at environmental temperature higher than 25°C, some newly emerged wasps (both PpNSRV-1(-) wasps and PpNSRV-1(+) wasps) were individually placed into tubes and reared at 35°C. The biological parameters were calculated as the following: successful parasitism rate (%) = number of host pupae parasitized by female wasps / total number of host pupae exposed to female wasps × 100; offspring number per parasitized pupa = number of emerged female or male wasps from the same parasitized pupa; offspring number per female wasp = number of emerged female or male wasps from all pupae parasitized by one female wasp; adult emergence proportion (%) = number of emerged adult wasps from a parasitized pupa / total number of emerged and unemerged adult wasps × 100; and offspring sex ratio (%) = number of female adult wasps emerged from a parasitized pupa / total number of female and male adult wasps emerged from a parasitized pupa × 100.

### Preparation of virus for microinjection assays

Crude virus liquid was prepared from adult wasps of a PpNSRV-1(+) colony as follows. Firstly, thirty ovaries from female wasps were dissected and placed into a 2-ml tube with 60 μl of PBS buffer (0.01 M, pH 7.4). Then a steel bead (2.5 mm in diameter) was placed into the tube, and the tube was shaken for 1 min at the speed of 25/s on a TissueLyser II (Qiagen, Maryland, USA). The homogenate was centrifuged at 12,000×*g* for 10 min at 4°C, and the resulting supernatant was subsequently filtered with a 0.22-μm sterile filter (EMD Millpore, Billerica, MA, USA). Virus-free crude liquid was prepared from the adult wasps of the PpNSRV-1(-) colony using the same method. The viral load of the liquid was quantified by qPCR. All samples were stored at -70°C.

To further confirm the effect of PpNSRV-1 infection on *P*. *puparum* wasps, we injected 50 nl of virus crude liquid (10^5^ copies/μl) into each yellow wasp pupa from the PpNSRV-1(-) colonies. The control was injected with an equal volume of virus-free crude liquid. The quantification of PpNSRV-1 in the wasps 3 days post-eclosion from injected wasp pupae was performed by qPCR. Then, we selected 30 injected female wasps and allowed them to have 48 h opportunity to mate to investigate the successful parasitism rate, adult emergence proportion, and offspring number. Newly emerged wasps [female: 34 PpNSRV-1(-), 35 PpNSRV-1 (+); male: 30 PpNSRV-1(-), 26 PpNSRV-1 (+)] from injected wasp pupae were individually placed into tubes and reared as described above for determination of longevity.

### Preparation of dsRNA and RNAi assays

To evaluate the effect of PpNSRV-1 on *P*. *puparum* wasps, we utilized RNAi knockdown of PpNSRV-1 ORF I to reduce the abundance of PpNSRV-1, and then evaluated effect of this knockdown on wasp fitness parameters. The ORF I-specific primers and primers targeting enhanced green fluorescent protein gene (eGFP; negative control) were designed with added T7 promoter adaptors (S2 Table). All amplified PCR products (400–600 bp) were cloned into pGEM-T easy vectors (Promega [Beijing] Biotech) and sequenced. The correct PCR products were used as templates for dsRNA synthesis with the MEGAscript T7 Transcription Kit (Ambion, Austin, TX), according to the manufacturer’s instructions. Synthesized dsRNA was purified by phenol/chloroform extraction and isopropanol precipitation, dissolved in diethylpyrocarbonate-treated water, and quantified using a NanoDrop 2000 Spectrophotometer (Nano-drop Technologies, Wilmington, DE) at 260 nm.

We injected 50 nl of dsRNA (2 × 10^3^ ng/nl) into each yellow wasp pupa from the PpNSRV-1(+) or PpNSRV-1(-) colony. The PpNSRV-1 titer in the wasps emerging from injected wasp pupae was quantified by qPCR. Then, 30 injected female wasps that had mated with injected male wasps for 48 h were selected to investigate the successful parasitism rate, adult emergence proportion, and offspring number. Newly emerged wasps were collected to measure longevity as described above.

### Statistical analysis

All values were expressed as mean ± standard error (S.E.M.). For qPCR and RNAi results, data were analyzed using a one-way ANOVA or two-way ANOVA followed by Tukey’s multiple comparison test (*p* <0.05). The means on each same measured biological parameter were compared between two colonies with and without carrying PpNSRV-1 using Student’s *t*-test (**p <0.01, *p <0.05). Linear regression analysis was constructed between viral detection rate and mean temperature from the five different cities in October, 2012, and the three various cities in June, 2016. Temperature was calculated based upon data from the Weather Underground website (https://www.wunderground.com). All statistical calculations were performed using Data Processing System (DPS) software (version 14.50) [75].

## Supporting information

S1 Fig Northern blot analysis of PpNSRV-1 ORFs I-V. (-) means virus-free and (+) means virus-infected.
(TIF)

S2 Fig Comparison of viral titer and adult longevity of *P*. *puparum* wasps at two different temperatures. (A) and (B) Viral load of PpNSRV-1 in adult wasps at 1, 3, 5, and 7 day post-eclosion reared at 25°C and 35°C respectively. Data represent means ± standard error (*n* = 3). Differences in viral load were not statistically significant (*t*-test). (C) Comparison on adult longevity between PpNSRV-1-free and PpNRSV-1-infected wasp colonies naturally reared at 35°C. (-) means virus-free and (+) means virus-infected. The number in column is the sample size for each treatment. Data represent means ± standard error. Statistical significance (*t*-tests) is indicated by asterisks: *, *p* < 0.05; **, *p* < 0.01.
(TIF)

S3 Fig Electron micrographs of purified PpNSRV-1 particles from follicular cells. (A) Electron micrographs of purified PpNSRV-1 virions. (B) and (C) are the magnification pictures. Red arrows indicated viral particles.
(TIF)

S4 Fig PCR detection and vertical transmission of PpNSRV-1. (A) Detection of PpNSRV-1 in individuals of different generations from virus-free and virus-infected wasp colonies. (-) means virus-free and (+) means virus-infected. The Arabic numbers represent the generation number. Vertical transmission of PpNSRV-1: (B) ♀+/♂+; (C) ♀−/♂−; (D) ♀+/♂− and (E) ♀−/♂+. M = marker (1 kb, 0.75 kb, 0.5 kb, 0.25 kb and 0.1 kb, respectively).
(TIF)

S5 Fig Comparison of total oocyte number in ovaries between PpNSRV-1-free and PpNRSV-1-infected wasp colonies. (-) means virus-free and (+) means virus-infected. The number in the columns is the sample size for each treatment. Data represent means ± standard error. Differences in oocyte number in the two colonies were not statistically significant (*t*-test).
(TIF)

S6 Fig Comparison of offspring among wild colonies and injected pupae. Comparison of offspring per parasitized pupa (female: A and C, male: B and D), offspring per female wasp (female: E and G, male: F and H) among wild colonies or injected pupae in each batch. (-) means virus-free and (+) means virus-infected. Batch means the experimental pupae of the same day. Data represent means ± standard error. Statistical significance (*t*-tests) is indicated by asterisks: *, *p* < 0.05; **, *p* < 0.01.
(TIF)

S7 Fig Viral load of PpNSRV-1 of *P*. *puparum* wasps that were injected with viral crude liquids. Viral load was determined in black pupae and adults at days 1 and 3 post-eclosion. Data represent means ± standard error (*n* = 3). SE bars annotated with the same letter are not significantly different (Tukey’s multiple comparison test).
(TIF)

S1 Table Viral sequences selected for phylogenetic analysis in this study. (DOCX)

S2 Table Primers used in this study. (DOCX)

S3 Table Detection of PpNSRV-1 in non-parasitized *P*. *rapae* pupae from different locations in China. (DOCX)
