# Supplementary material for: A novel negative-stranded RNA virus mediates sex ratio in its parasitoid host
Source: PLoS Pathog. 2017 Mar 9;13(3):e1006201. doi: 10.1371/journal.ppat.1006201 (PMC5344506; doi:10.1371/journal.ppat.1006201)
Supplement: S1 Table — (DOCX) [file ppat.1006201.s008.docx]

**S1 Table. Viral Sequences Selected for Phylogenetic Analysis in This Study**

| **Mononegaviral family** | **Genus** | **Natural host** | **Virus** | **Virus abbreviation** | **RefSeq or GenBank accession number** |
| --- | --- | --- | --- | --- | --- |
| *Bornaviridae* | *Bornavirus* | Horses; sheep; cattle; rodents; birds; humans | Borna disease virus 1 | BoDV-1 | NC_001607 |
|  |  |  | Parrot bornavirus 1 | PaBV-1 | GU249595 |
|  |  |  | Canary bornavirus 1 | CnBV-1 | KC464471 |
|  |  |  | Aquatic bird bornavirus 1 | ABBV-1 | NC_029642 |
| *Filoviridae* | *Cuevavirus* | Unknown | Lloviu virus | LLOV | NC_016144 |
|  | *Ebolavirus* | Unknown | Ebola virus | EBOV | NC_002549 |
|  | *Marburgvirus* | Bats | Marburg virus | MARV | NC_001608 |
| *Mymonaviridae* | *Sclerotimonavirus* | Fungi | Sclerotinia sclerotiorum negative-stranded RNA virus 1 | SsNSRV-1 | NC_025383 |
| *Nyamiviridae* | *Nyavirus* | Ticks; birds | Nyamanini virus | NYMV | NC_012703 |
|  |  |  | Midway virus | MIDWV | NC_012702 |
|  |  |  | Sierra Nevada virus | SNVV | NC_024376 |
|  | *Socyvirus* | Nematodes | Soybean cyst nematode virus 1 | SbCNV-1 | NC_024702 |
| *Paramyxoviridae* | *Aquaparamyxovirus* | Fish | Atlantic salmon paramyxovirus | AsaPV | EF646380 |
|  | *Avulavirus* | Birds | Newcastle disease virus | NDV | NC_002617 |
|  | *Ferlavirus* | Reptiles | Fer-de-Lance virus | FDLV | NC_005084 |
|  | *Henipavirus* | Bats; humans | Hendra virus | HeV | NC_001906 |
|  | *Morbillivirus* | Humans; dogs; cats; cetaceans | Measles virus | MeV | NC_001498 |
|  | *Respirovirus* | Rodents; humans | Sendai virus | SeV | NC_001552 |
|  | *Rubulavirus* | Humans; apes; pigs; dogs | Mumps virus | MuV | NC_002200 |
| *Pneumoviridae* | *Metapneumovirus* | Humans; birds | Avian metapneumovirus | AMPV | NC_007652 |
|  | *Orthopneumovirus* | Humans; cattle; rodents | Human respiratory syncytial virus | HRSV | NC_001781 |
| *Rhabdoviridae* | *Cytorhabdovirus* | Plants; insect vectors: aphids, leafhoppers, planthoppers | Lettuce necrotic yellows virus | LYNV | NC_007642 |
|  | *Dichorhavirus* | Plants | Orchid fleck virus | OFV | NC_009609 |
|  | *Ephemerovirus* | Cattle; mosquitos | Bovine ephemeral fever virus | BEFV | NC_002526 |
|  | *Lyssavirus* | Human; mammals | Rabies virus | RABV | NC_001542 |
|  | *Novirhabdovirus* | Fish | Infectious hematopoietic necrosis virus | IHNV | NC_001652 |
|  | *Nucleorhabdovirus* | Plants; insect vectors: planthoppers and aphids | Potato yellow dwarf virus | PYDV | NC_016136 |
|  | *Perhabdovirus* | Fish | Perch rhabdovirus | PRV | NC_020803 |
|  | *Sigmavirus* | Fruit flies | Drosophila melanogaster sigmavirus | DMelSV | NC_013135 |
|  | *Sprivivirus* | Fish | Spring viraemia of carp virus | SVCV | NC_002803 |
|  | *Tibrovirus* | Cattle; water buffalo | Tibrogargan virus | TIBV | NC_020804 |
|  | *Tupavirus* | Birds | Durham virus | DURV | FJ952155 |
|  | *Varicosavirus* | Plants | Lettuce big-vein associated virus | LBVaV | NC_011558 |
|  | *Vesiculovirus* | Humans; cattles; horses; swines; sandflies; blackflies | Vesicular stomatitis Indiana virus | VSIV | NC_001560 |
| *Sunviridae* | *Sunshinevirus* | Snakes | Sunshine Coast virus | SunCV | NC_025345 |
