# Supplementary material for: A novel negative-stranded RNA virus mediates sex ratio in its parasitoid host
Source: PLoS Pathog. 2017 Mar 9;13(3):e1006201. doi: 10.1371/journal.ppat.1006201 (PMC5344506; doi:10.1371/journal.ppat.1006201)
Supplement: S2 Table — (DOCX) [file ppat.1006201.s009.docx]

**S2 Table. Primers Used in This Study**

| **Primer** | **Genome position** | **Oligonucleotide (5'→3')** | **Orientation** | **Application** |
| --- | --- | --- | --- | --- |
| VS-1 | 90-111 | GGGGCATAGGGACAAGTTTTCT | → | To confirm viral genome |
| VA-1 | 1537-1558 | CTTTGCCCTTGTGATGTGATTG | ← |  |
| VS-2 | 1150-1171 | TCACATTCACATTATCGCTCCC | → |  |
| VA-2 | 2459-2480 | ACACTTTGGACCGAAGATTACC | ← |  |
| VS-3 | 2373-2394 | TTGAGGAAGTTGACTAAGGGAG | → |  |
| VA-3 | 3835-3856 | CTTCGACTGGCTCAAGAGTTAC | ← |  |
| VS-4 | 3620-3641 | AGCAATCCTCCTGAAGAAATAC | → |  |
| VA-4 | 5026-5047 | CAGATACTGCCTCTATTCAAGC | ← |  |
| VS-5 | 4949-4970 | AGTGTCTGTTTCAGGCAGTGTA | → |  |
| VA-5 | 6564-6586 | GCCCTTCAGACAGATAAGATAGA | ← |  |
| VS-6 | 6250-6271 | TTCCGCACTCCTTCAGTTCTTT | → |  |
| VA-6 | 7608-7631 | TTGTGGTCTCAGAGTGTCGGTTAC | ← |  |
| VS-7 | 7358-7379 | GGTAAGAGTGTAAGCCGCAAGA | → |  |
| VA-7 | 8650-8671 | GGGGCTTCATAACTGACTCTTC | ← |  |
| VS-8 | 8328-8345 | CAGTCATAGCCGACCAAA | → |  |
| VA-8 | 9982-10003 | AATGGACAAGGGTCTAACTGAT | ← |  |
| VS-9 | 9596-9616 | GAGTCTTGGCGTCCTTCTTCC | → |  |
| VA-9 | 11149-11170 | TCGGACTACCAGAGGGACCACG | ← |  |
| VS-10 | 10880-10898 | TGAGCACACAACCCCATGA | → |  |
| VA-10 | 12206-12230 | CAAAAGATGAAAATCCTCTGTAACA | ← |  |
| RS-1 | 11760-11782 | CAACAAGAATCCCCGTGTAATCC | → | Termini determination |
| RS-2 | 11858-11880 | AACTGGGCTCCATCAACTCTCGG | → | of genome |
| RS-3 | 11928-11950 | TAGCTCGGAACGAGATCAGGTCC | → |  |
| RS-4 | 12010-12032 | CTGAAGGAGCGGATTTAGGTCGG | → |  |
| RA-1 | 648-671 | TCAACTCTTATGTGCTCTGGTCGC | ← |  |
| RA-2 | 452-475 | ACGAATCTACTGGTGCCTGGGAAC | ← |  |
| RA-3 | 312-335 | AATACCATTGCATCCCTCTAAGGC | ← |  |
| RA-4 | 198-221 | GAATAAGATCCAGCCAAGAAGTGG | ← |  |
| O1-RS-1 | 11785-11808 | ATCCGTGCGTTTGAGAACAGTGGC | → | 5' RACE of ORF I |
| O1-RS-2 | 11665-11688 | ATTGAGCTCGTCCTCATTGGCGAG | → |  |
| O1-RS-3 | 11519-11542 | ACCAATGCCGTACACACCCGACTC | → |  |
| O1-RA-1 | 10303-10326 | GCCCTTCCATCTGCACCAAGATCA | ← | 3' RACE of ORF I |
| O1-RA-2 | 10227-10250 | CTGCCATTAGAACCGATGATGCCA | ← |  |
| O1-RA-3 | 10535-10558 | TGGTGCCTGGGCTATGGATCAGAT | ← |  |
| O2-RS-1 | 10005-10028 | CTTCTTCATGACCGGCTGCTGTTG | → | 5' RACE of ORF II |
| O2-RS-2 | 9813-9836 | CTTTGAGATCGTTGGCCTCCTGCA | → |  |
| O2-RS-3 | 9761-9784 | TTTGGACGGACGATCTCTTTTGCA | → |  |
| O2-RA-1 | 9823-9846 | GACACTCTCCTGCAGGAGGCCAAC | ← | 3' RACE of ORF II |
| O2-RA-2 | 10097-10120 | GAGCACTCCACACCCATCCACATC | ← |  |
| O2-RA-3 | 10005-10028 | CAACAGCAGCCGGTCATGAAGAAG | ← |  |
| O3-RS-1 | 9120-9143 | GCCCTTGGGAACTGCTCCACTCTC | → | 5' RACE of ORF III |
| O3-RS-2 | 9218-9241 | TCTGTTCCCTGGTTGCTGTCCTGC | → |  |
| O3-RS-3 | 9384-9407 | TGGATCACGGGTCTCATCTGGCTC | → |  |
| O3-RA-1 | 8760-8783 | GCAGGAGACCGATTGACCAAGGCT | ← | 3' RACE of ORF III |
| O3-RA-2 | 8666-8689 | GACAGGCTGCTGCTAGTAGGGGCT | ← |  |
| O3-RA-3 | 8836-8859 | GAAATCATCGAAGGGCACAGCAGG | ← |  |
| O4-RS-1 | 7994-8017 | GGTAACTCCTCCTGGGTTCACCGA | → | 5' RACE of ORF IV |
| O4-RS-2 | 8188-8211 | TCTTGTGAGGGGTCTGGGTCTGCT | → |  |
| O4-RS-3 | 7740-7763 | TGCCAGGAGCACATTGCTGTAGGA | → |  |
| O4-RA-1 | 7378-7401 | ATCAGGCAAGCAAGCATCCAGGTC | ← | 3' RACE of ORF IV |
| O4-RA-2 | 7150-7173 | CCATTGAGTGCGATGACGATTCCA | ← |  |
| O4-RA-3 | 7262-7285 | GTTCTGCGGCCACAGGATGAGTGT | ← |  |
| O5-RS-1 | 5884-5907 | CTCCTACGCAAATGGGTTCCAAGC | → | 5' RACE of ORF V |
| O5-RS-2 | 6126-6149 | TGAAGAACCACAAGGTCCCCCACT | → |  |
| O5-RS-3 | 6452-6475 | CCAAGGAAGAACAGGAGTGGCGTC | → |  |
| O5-RA-1 | 383-406 | GGATGGGACAAGTGCTGCCTGAAG | ← | 3' RACE of ORF V |
| O5-RA-2 | 687-710 | GCAGACTTCACCATCTCCCAAGGC | ← |  |
| O5-RA-3 | 441-464 | GGTGCCTGGGAACTTCATGGTAGC | ← |  |
| O1-NBS | 10688-10707 | GATGGCAAGATATCCACTAA | → | Northern blot |
| O1-NBA | 10994-11013 | TTCATTCTTCCTGTCTTTGA | ← |  |
| O2-NBS | 9713-9732 | AATCTGAATGTGATGGCTAT | → |  |
| O2-NBA | 9996-10015 | TCATGAAGAAGAAATGGACA | ← |  |
| O3-NBS | 8959-8978 | AGACACCACAAATAATGAGT | → |  |
| O3-NBA | 9303-9322 | TTGAGGTAATTGAGAGTGAC | ← |  |
| O4-NBS | 7723-7742 | CTCCTTGGTATATACTCTGC | → |  |
| O4-NBA | 8082-8101 | GACTTAACCCCATACATCAT | ← |  |
| O5-NBS | 2650-2669 | CTATCACTGCACTCAAAATC | → |  |
| O5-NBA | 2974-2993 | AACTTCCAATAGAGCTACAG | ← |  |
| QVA-1 | 6243-6262 | AAGGAGTGCGGAAGAGACAA | ← | Quantification of PpNSRV-1 |
| QVS-1 | 6097-6116 | TGATGATCACTGGCCCTGTT | → |  |
| VDA-1 | 5026-5047 | CAGATACTGCCTCTATTCAAGC | ← | Detection of PpNSRV-1 |
| VDS-1 | 4542-4563 | TCACTGAGTTGTCGTTTGATAG | → |  |
| VDA-2 | 1152-1171 | GGGAGCGATAATGTGAATGT | ← |  |
| VDS-2 | 648-667 | GCGACCAGAGCACATAAGAG | → |  |
| ABVA | 6391-6411 | ATAACATTGAAGACATTTGGC | ← | Template for standard |
| ABVS | 5876-5895 | CAATACAACTCCTACGCAAA | → | curve |
| VORF1A | 12058-12078 | CGAAGGTACAACTACAGGCAT | ← | Prokaryotic expression |
| VORF1S | 10216-10235 | CTACTGCTCCATGGCATCAT | → |  |
| T7ORF1-1A | 10994-11013 | TTCATTCTTCCTGTCTTTGA | ← | Synthesis of dsRNA |
| T7ORF1-1S | 10484-10503 | TTGACTCCACTTTCTATCAG | → |  |
| T7ORF1-2A | 10849-10868 | AGTACCACATCAAGAATTGT | ← |  |
| T7ORF1-2S | 10338-10357 | CTGTCAGATTGGAGAACATA | → |  |
| T7eGFPA |  | AAGGGCGAGGAGCTGTTCACCG |  |  |
| T7eGFPS |  | CAGCAGGACCATGTGATCGCGC |  |  |
| T7promotor |  | TAATACGACTCACTATAGG |  |  |
| QPp-18S-SP | | CGGGGAGGTAGTGACGAA |  |  |
| QPp-18S-AP | | CGAGCGATGAACCGACAG |  |  |
