# Supplementary material for: A novel negative-stranded RNA virus mediates sex ratio in its parasitoid host
Source: PLoS Pathog. 2017 Mar 9;13(3):e1006201. doi: 10.1371/journal.ppat.1006201 (PMC5344506; doi:10.1371/journal.ppat.1006201)
Supplement: S3 Table — (DOCX) [file ppat.1006201.s010.docx]

**S3 Table. Detection of PpNSRV-1 in Non-parasitized *P. rapae* Pupae from Different Locations in China**

| **Locations** | **Sampling time** | **Number of non-parasitized *P. rapae* pupae sampled** | **Percentage of *P. rapae* pupae harboring PpNSRV-1 (%)** |
| --- | --- | --- | --- |
| Hefei (117.16°E, 31.31°N) | October, 2012 | 106 | 0 |
|  | June, 2016 | 95 | 0 |
| Hangzhou (120.19°E, 30.26°N) | October, 2012 | 110 | 0 |
|  | June, 2016 | 98 | 0 |
| Jiande (119.27°E, 29.49°N) | October, 2012 | 100 | 0 |
|  | June, 2016 | 105 | 0 |
| Ningbo (121.56°E, 29.86°N) | October, 2012 | 110 | 0 |
| Nanchang (115.53°E, 28.41°N) | October, 2012 | 98 | 0 |
